# Some Cars for Professional Men

**Published:** 1920-11-13

**Authors:** 


### THE MOTOR SHOW.

### Some Cars for Professional Men.

To those in the motor trade the fact that this year's Show has been not only absolutely international but also established, by its magnitude, London as *the* motor mart of the world may be accepted with profound satisfaction. But we are not so sure that those professional men who have snatched a moment from their labours to see the exhibits for themselves will appreciate the enlargement in the same degree. The appalling congestion of last year's Olympia may have been relieved, but to those for whom time is valuable, to the modified crush was added the extra space to be covered, and the change cannot be described as helpful. It is probable, therefore, that few who read these notes will, from the 459 types exhibited, have had more than a small chance of making a satisfactory judgment thereon. Let us, therefore, summarise, in the first place, what the average professional was best advised to seek and, in the second, what there was to be found.

Rumours of general price-cutting in the automobile industry are an absolute myth—for the present, at any rate. A few of the machines with high rate of output facilities behind them have been able to come down considerably, but the majority, particularly the higher-powered cars, are prices distinctly higher than at last year's Show, and it looks as though he who is waiting for prices in general to fall will wait a considerable time.

On the other hand, this year it was actually possible to buy a machine at the Show, and that with a reasonable hope of seeing it in one's own garage before the New Year. Apparently production is now on a scale adequate, perhaps more than adequate, to meet the demands, which situation should indicate to the doctor a most important guiding principle in his choice. There are a number of new firms turning out highly attractive vehicles of the correct h.p. and price, but, uncharitable though it may sound to a new and worthy venture, we do not entirely rely on the repair and spare-part service which a hard-run doctor's car will obtain from these companies in two or three years' time. The grounds for such fears are fairly obvious, so we will not labour them; but they lead us to a firm decision that our choice for the immediate future at least, shall fall among those makes already soundly established before the war.

As regards the size and weight of the professional car; running expenses have soared out of all proportion to the actual cost of the vehicle. On January 1, too, the new tax of £1 per horse-power to the motorist is in sad and obvious contrast to that of last year.

On these lines, therefore, it is safe to take a 20 h.p. Treasury rating as the outside limit in size for average professional use, and at this point it is only fair to mention the mass-production Austin car. This machine is already well known to most of us. Also we have all heard scathing criticism of the "finish" of its exterior, panelling, etc. But suffice it to say that the general turn-out of the Austin is far ahead of the similar-priced American, and it is by now a fully proved and essentially serviceable car. The same thing can be claimed for the 14-h.p. Angus-Sanderson, which represents extraordinarily good value at £575. That is, after all, what the doctor wants. It is true he will find more elaborate models at this power—the new 19.6-h.p. Crossley, the 16-h.p. Sunbeam, the 15-h.p. Wolseley, are typical examples—but to buy these he will have to increase his initial outlay considerably.

On the whole, with money at its present scarcity, we feel that there is little doubt that the doctor's car of the moment is the 12 h.p., and this because these machines come just within the owner-driver category; they can be truthfully said to exceed twenty miles to the gallon of petrol, and their weight and size allow of reasonably low repair and spare-part costs. In the outline description which follows we shall include sundry slightly smaller models, but the chief aim will be to mention, and that quite disinterestedly, those machines that, to the practising profession, we think are really worthy of consideration.

Sundry forecasts of the Show selected the 12-h.p. Rover as an outstanding vehicle of its type. We agree that it is excellent, well up to pre-war standard, turned out complete in every way, and improved in full accord with the demands and progress of the times. It is not cheap, but is, nevertheless, good value for money at the moment, and, like its predecessor, should do good service for years. More novel is its younger brother, the air-cooled, horizontal, twin-cylinder 8-h.p. Rover, which has already distinguished itself in competitions, and, together with a number of other small air-cooled cars, may be taken as typical of a "small" class which has come to stay. They are too small, perhaps, for average professional use, but their economy in running, the simplicity of the engine mechanism, and the fact that they cannot freeze, however long they wait outside a house in the winter, give them an obvious claim on the attention of many country practitioners. Emphatically these little cars are not experimental, and can be ordered with the greatest confidence when turned out by a firm of good reputation.

The next two models which we personally noticed were the Standards (9.5 h.p. and 11.6 h.p.). They are both exceptionally well equipped cars, and their square lines allow of particularly neat and serviceable arrangement of the hood and side windows, so that the open models can in truth be converted into closed all-weather models without the bother usually associated with this performance, or the extra expense of a recognised *coupé* model. There is also ample room for the passengers and their legs, a provision many of the newer makers seem entirely to have overlooked. The 12-h.p. Swift, too, is a satisfactory machine in most ways. Its lines are quite orthodox, and its coachwork particularly good. The most important feature in connection with it is the fact that it comes among the small band whose prices *have* fallen since last year. Professional men do not need to be reintroduced to the Singer light car, the smaller Humbers, or the Morris Cowley. All have been modernised, they are better turned out than last year, fault with them is hard to find, and a purchaser can make his choice with equal reliance on any of them.

Of the smaller foreign cars, the most remarkable for performance and for actual value, though not, indeed, for finish, is the French 10-h.p. Citroen. A trial run in a new machine is certainly a revelation, but one is sceptical as to how long it will stand hard wear on modern "roads." The spare-part service seems to be genuinely assured, however, the car is absolutely complete, and the initial price is low, so that this machine is well worthy of close inspection. Among others not to be missed were the 15-h.p. Metallurgique; the Overland, of Crystal Palace steps fame; the Calthorpe *coupés*; the new Charron-Laycock, which tried to appear last year, but was forced by labour troubles to wait; the particularly smart-looking A.C. two-seaters; and the Hillman. The 11.9-h.p. Beau has received enough Press attention already; it is a remarkable car at a remarkably low price. But, lest we forget, though the Ford was not in the Show, we saw its exhibition near by. It is quite inadequate to say that this astonishing car is more incredibly good value than ever.

